# Detecting Arsenic Contamination Using Satellite Imagery and Machine Learning

**DOI:** 10.3390/toxics9120333

**Published:** 2021-12-03

**Authors:** Ayush Agrawal, Mark R. Petersen

**Affiliations:** 1 Canyon Crest Academy, San Diego, CA 92130, USA; 2 Computational Physics and Methods Group, Los Alamos National Laboratory, Los Alamos, NM 87545, USA; mpetersen@lanl.gov

**Keywords:** remote sensing, machine learning, imaging spectroscopy, environmental contamination, land cover, satellite imagery, dimensionality reduction, arsenic detection, EO-1 Hyperion

## Abstract

Arsenic, a potent carcinogen and neurotoxin, affects over 200 million people globally. Current detection methods are laborious, expensive, and unscalable, being difficult to implement in developing regions and during crises such as COVID-19. This study attempts to determine if a relationship exists between soil’s hyperspectral data and arsenic concentration using NASA’s Hyperion satellite. It is the first arsenic study to use satellite-based hyperspectral data and apply a classification approach. Four regression machine learning models are tested to determine this correlation in soil with bare land cover. Raw data are converted to reflectance, problematic atmospheric influences are removed, characteristic wavelengths are selected, and four noise reduction algorithms are tested. The combination of data augmentation, Genetic Algorithm, Second Derivative Transformation, and Random Forest regression ($R^{2}=0.840$ and normalized root mean squared error (re-scaled to [0,1]) = 0.122) shows strong correlation, performing better than past models despite using noisier satellite data (versus lab-processed samples). Three binary classification machine learning models are then applied to identify high-risk shrub-covered regions in ten U.S. states, achieving strong accuracy (=0.693) and F1-score (=0.728). Overall, these results suggest that such a methodology is practical and can provide a sustainable alternative to arsenic contamination detection.

## 1. Introduction

Contamination due to heavy metal pollution has emerged as one of the leading environmental concerns in the last few decades, causing a variety of adverse effects on both human and overall ecosystem health. Human activities such as poor waste management, fossil fuel exploitation, and excessive mining of metal ores release arsenic (As) into soil and groundwater [1], from where it can easily spread throughout the environment. In 2020, an estimated 220 million people worldwide were exposed to arsenic concentrations that exceeded recommended limits [2,3]. Densely-populated Asian countries such as China, India, Cambodia, Pakistan, and Vietnam are among the most severely impacted, but detrimental arsenic contamination has also been found in large concentrations in Argentina and parts of the United States [3].

Arsenic is found abundantly in both the Earth’s crust and on the surface [4]. Past medical research and data show that inorganic arsenic exposure can cause many different types of cancer such as lung cancer [5], leading to its classification as a Group 1 human carcinogen [6]. Published literature further indicates that arsenic is toxic to human neural development: consuming arsenic has been linked with oxidative stress, which interferes with the biological ability to detoxify accumulating oxygen reactive species and potentially contributes to aging, neurogenerative diseases, and cancer [7]. Inorganic arsenic can easily increase risks of pregnancy outcomes such as impaired fetal growth [8]. Arsenic poisoning also has many economic implications as it affects agriculture, water management, and public health [9]. Coupled with its mobility in the environment as part of natural biogeochemical cycles, arsenic poses a serious threat to human health. Traditional approaches to arsenic contamination detection involve two methods: lab-based techniques and colorimetric field tests.

Lab-based techniques, primarily involving High-Performance Liquid Chromatography (HPLC), Inductively Coupled Plasma Mass Spectrometry (ICP-MS), Optical Emission Spectroscopy (OES), and Atomic Absorption Spectrometry (AAS), have been the traditional way to detect arsenic since the 1990s, with advances in technology allowing for more precise detection of arsenic [10]. However, even with the significantly cheaper costs of the OES and AAS instruments due to trade-offs in their detection limits, the cost of using any of these lab-based spectral technologies is in the thousands of dollars [10]. These instruments are also known to be laborious and unportable due to their design. Furthermore, as arsenic is often distributed unevenly throughout an area, known as spatial heterogeneity [11,12], laboratory methods for one area are often not accurately applicable to other regions.

Methods directly implementable in the field include the Gutzeit method, which works by forming a color complex when arsine gas (AsH3) reacts with mercuric bromide, with the intensity of the color being proportional to the arsenic concentration in the water sample [13]. However, the interpretation of this color change requires the human user to rely on accurate visual perception, making it difficult to operate without training. In addition, researchers have found the results to differ noticeably from the more accurate lab test results [13]. One study found that the kits gave 68% false negative results and 35% false positive results, with 44.9% of samples found to have been mislabeled, especially those below the upper safety limit of arsenic [14]. Furthermore, even if the cost of one test is cheap, applying tests multiple times to hundreds of sites can amount to high costs.

Recent advances in remote sensing and machine learning can make a more scalable and low-cost solution possible. Hyperspectral remote sensing is an emerging field that uses imaging spectroscopy to obtain images in continuous spectral ranges from 0.4 to 14 µm [15]. Compared to its predecessor, multispectral imaging, hyperspectral imagery offers a variety of advantages, including high spatial resolution and an appreciably larger amount of spectral information [16]. Hyperspectral imagery has been used to remotely study chemical and physical properties of the geology of a given region, with application to fields such as precision agriculture, ecological modeling and monitoring, discovering important ores, and urban planning [17]. NASA’s EO-1 Hyperion satellite, which orbited the Earth daily from 2001 to 2017 and gathered over 54 terabytes of data in the process, pioneers important use of this technology. Hyperion provides high spectral resolution data with 242 bands at 10 nanometer (nm) spectral resolution, 30 m length pixels, and swath width of 7.5 km [18], collecting detailed images (in the form of 3D-cubes) over a large geographical area relatively efficiently.

Multiple research studies have been conducted in the past few years to explore relationships between hyperspectral data and heavy metal contamination in bare soil, including As, Chromium (Cr), Cadmium (Cd), Lead (Pb), and Zinc (Zn), using regression-based machine learning approaches on small-field scales with lab-processed soil data and hyperspectral measurements [11,12,18,19,20,21,22,23,24,25,26,27]. This approach is advantageous because it offers a remote yet effective technique for scalable heavy metal detection. Most of these studies indicate reasonable success with similar methodologies across all the mentioned heavy metals. For example, when detecting arsenic, Zhang et al. [20] used a GA-enhanced machine learning model to achieve 35.0% correlation between the two variables, and Liu et al. [22] used a Particle Swarm Optimized (PSO) Back Propagation Neural Network (BPNN) to achieve 81.1% correlation.

A lesser number of studies have been conducted using airborne sensors and multispectral satellites [28,29,30]. Peng et al. [28] studied bare Qatari soils and were able to detect a variety of heavy metals, including arsenic, using multispectral imagery from Landsat 8 with good accuracy. However, multispectral imagery offers less spectral information than hyperspectral imagery. The Landsat satellite itself also has limitations due to slow repeated global coverage [31]. While Choe et al. [30] studied heavy metals using the hyperspectral HyMAP, they used an airborne sensor that comes with low area coverage and high operational costs [18]. Both are significant disadvantages in large-scale monitoring, especially when compared to the use of Hyperion.

To the author’s knowledge, no published study has yet used hyperspectral data directly from an orbiting satellite besides a cadmium-focused implementation of the HJ-1A satellite that did not yield promising results for arsenic [32]. Furthermore, no published study has tested applicability to shrub-covered regions nor applied a classification approach over a large-field scale. In this study, it was hypothesized that data from Hyperion combined with a regression-based machine learning approach on bare soil would yield strong correlation and comparable results to the best models (using less noisy lab-based data) used in literature ($R^{2}>0.7$). Second, it was hypothesized that high-risk shrub-covered soil could be identified with reasonable metrics using binary classification. Third, it was hypothesized that the application of spectral preprocessing, data augmentation, and dimensionality reduction would serve to increase the accuracies of the baseline models. Note that dimensionality reduction has some disagreement in literature regarding its effectiveness and necessity; while some studies’s models benefit from it [21], some have found it ineffective [26].

The results indicated that all three of the above hypotheses were met, with the DA + GA + SD + RF regression model achieving $R^{2}=0.840$ and the Multi-Layer Perceptron (MLP) binary classification model achieving an average accuracy =0.693 and F1-score =0.728. Overall, the goal of this study was able to identify a simple, versatile, and effective approach that can provide a low-cost, rapid, less labor-intensive, and still accurate alternative to arsenic contamination detection, which was successfully accomplished.

## 2. Materials and Methods

**Figure 1:**
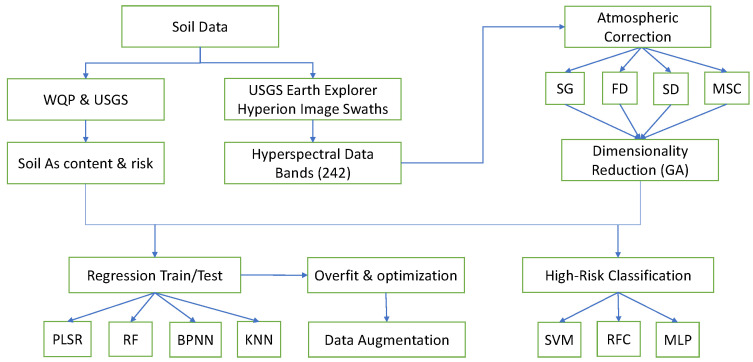
Workflow diagram for arsenic detection from hyperspectral satellite data. WQP stands for the Water Quality Portal and USGS is the United States Geological Survey. Other abbreviations are listed at end of the article.

The primary objective of this research is to determine whether a correlation can be determined between hyperspectral satellite data and soil arsenic concentration, with the secondary goal of extending this to a new land cover and classification approach if the first is successful. All data were obtained from publicly available resources and all algorithms were coded in Python. All work was done over the course of a year using a standard home laptop, further illustrating the power and versatility of the method. A flowchart illustrating all of the major steps is diagrammed in Figure 1.

### 2.1. Data Acquisition

**Figure 2:**
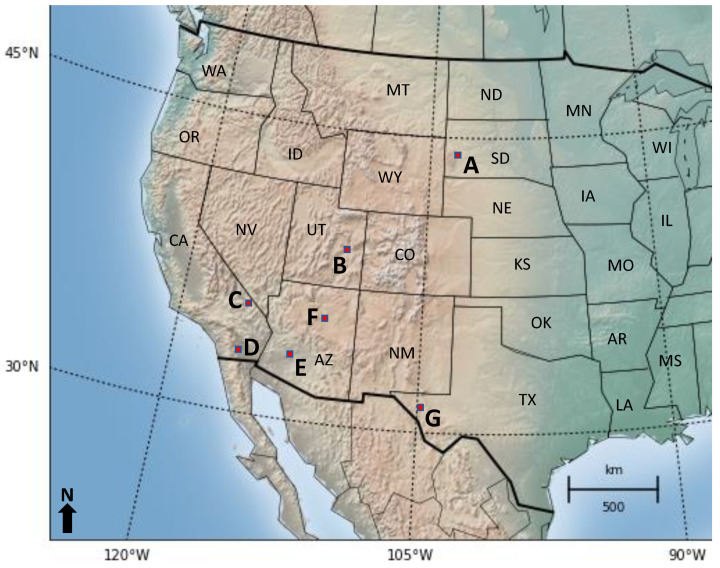
Map of the Western United States showing the locations of the 55 locations picked as part of bare soil regression validation dataset in this study. Data were chosen from USGS sites in sub-regions A–G (indicated with red squares), with the specific locations in each sub-region shown in Figure 3.

Publicly available data for soil arsenic concentration in the United States between 2005 and 2020 for both aforementioned land covers (in mg/kg) were processed for the top layer of soil (0–5 cm depth). After cross-referencing with availability of EO-1 Hyperion’s hyperspectral data, soil arsenic data from the regions highlighted in Figure 2 were selected for bare soil regression (the data used for shrub land classification is described later). A total of 55 bare soil arsenic concentration values, with concentrations between 1.4 mg/kg and 380 mg/kg, were used for regression analysis. Hyperion hyperspectral data were downloaded as rectangular image swaths. The actual hyperspectral values were taken from the 30 m by 30 m subplot that contained the selected coordinate. All coordinates chosen were identified to be in different subplots in order to prevent overlap.

### 2.2. Geometric and Atmospheric Corrections

Preprocessing of the hyperspectral satellite data is necessary to reduce noise and data redundancy, reducing the error caused by external influences.

Geometric correction involves distortions caused by errors such as the satellite’s positioning on its orbit and angle changes due to Earth’s rotation. NASA performs most base-level geometric processing before releasing Hyperion datasets publicly [33]. NASA’s provided datasets contain hyperspectral values in a 2D format, with swaths angled away from geographic north while measuring regions (due to Earth’s rotation). Given the latitude-longitude coordinates of the four corners, a linear interpolation approach was used to determine the specific swath pixel containing the desired latitude-longitudinal location. As the geographical scale of each individual swath (averaging approximately 1.3 degrees of latitude and 0.4 degrees of longitude) is relatively small, linear interpolation provides very reasonable approximations.

Next, to correct for problematic atmospheric conditions, the default numerical radiance values were converted into reflectance values using the well-known formula [34]:

$$\rho \rho =\frac{\pi L_{\lambda }d^{2}}{cos\theta_{s}\cdot {ESUN}_{\lambda }},$$

where ρρ is reflectance, $L_{\lambda }$ is the spectral radiance at the sensor’s aperture, *d* is the Earth–Sun distance in astronomical units (AU), ${ESUN}_{\lambda }$ is the mean solar exo-atmospheric irradiance (provided by NASA), and $\theta_{s}$ is the solar inclination angle in degrees. As reflectance measures the ratio of the amount of light leaving a target location to the amount of light striking the target, the conversion helps minimize the effects of atmospheric scattering, varying Earth–Sun distances, and solar elevation at time of measurement.

Next, potentially troubling Hyperion bands are identified and filtered out based on past literature and basic atmospheric principles [34,35,36]. The Hyperion Visible-Near Infrared (400–1100 nm) sensor makes up 70 of the 242 hyperspectral bands, with the other 172 composed from the Short-wave Infrared (1100–2500 nm) sensor. Due to low sensitivity regions and overlap between the two sensors, bands 1 to 7, 58 to 78, and 224 to 242 are removed from the dataset. Atmospheric water vapor bands, which absorb almost all relevant solar radiation, and bands likely to contain unstable atmospheric effects are further removed. The final subset consists of 155 bands: 10 to 57 (448–926 nm), 81 to 97 (953–1114 nm), 101 to 119 (1155–1336 nm), 134 to 164 (1488–1790 nm), and 182 to 221 (1972–2365 nm).

A concern with using satellite-based hyperspectral imagery as opposed to lab-based spectrometers is that the satellite might be picking up on a factor such as geology or land/ground cover (shrubland, forest, bare ground, urban/suburban, etc.) as opposed to the actual differences in arsenic concentration. Spectra for regions that have similar land cover and overall soil composition but differences in arsenic concentration were first visually compared to investigate if this was occurring; the results of this analysis are presented in Section 3.1 of this paper (‘Land Cover Verification’).

### 2.3. Spectral Preprocessing

For the final preprocessing step, four different spectral preprocessing methods were tested, each having its distinct advantages and successes in past research [19]: Savitzky–Golay (SG), First Derivative Transformation (FD), Second Derivative Transformation (SD), and Multiplicative Scatter Correlation (MSC). SG smoothing allows for the improvement of the accuracy of noisy synthetic data without losing important baseline trends [37,38]. FD can improve data stability. SD magnifies smaller differences between wavelengths and reduces some sources of random error caused by external factors through this focus on differences. MSC can correct for certain errors caused by light scattering [26].

For the purposes of this study, each of the SG, FD, and SD filters use a window length (the number of coefficients) of 17 and a polynomial order/degree of 4, as consistent with past hyperspectral research [18,26]. The predictive ability of these four noise reduction algorithms was tested by combining their processing of the spectra with the PLSR ML model. $R^{2}$ and nRMSE values of each model were compared against the other transformations and the main models, as explained later.

### 2.4. Regression Analysis with Bare Soil Regions

#### 2.4.1. Machine Learning Models

After preprocessing, the dataset was used for establishing baseline correlations using four machine learning models: Partial Least Squares Regression (PLSR), Back Propagation Neural Network (BPNN), Random Forest (RF), and K-Nearest Neighbors (KNN). While PLSR is the most common regression algorithm for hyperspectral data [39], it has been noted in previous studies that there likely exists a complex nonlinear relationship between hyperspectral data and arsenic content [40], which is why the nonlinear models—BPNN, RF, and KNN—were added.

PLSRs are used frequently because they maximize the correlation of the extraction of principal components from variables [21]. BPNNs are efficient models that first process information forwards and then iterate on their errors by going backwards, repeating the process until best possible optimization [41]. (For this study, all BPNN models consist of an input layer with 155 input neurons, 1 hidden layer with 7 neurons, and an output layer with 1 neuron. The Tangent Hyperbolic function (tanh) was used as the activation function.) Random Forest (RF) regression splits a dataset into multiple subsets that it then attempts to fit its decision trees on, eventually recombining the subsets through averaging, helping minimize overfitting and outlier influence [42,43]. K-Nearest Neighbors Regression (KNN) “predicts its target based on local interpolation of the targets associated with the nearest neighbors in the training set” [42].

The performances of the machine learning models were evaluated using the coefficient of determination ($R^{2}$), measuring the variance the predicted data have to the generated regression line, and normalized root mean square error (nRMSE), measuring the standard deviation of the prediction errors divided by the highest value of the dataset [44]. All regression models and their relevant parameters were optimized (Table 1). To test the predictive ability of the model, the dataset was split into a training group of 70% and a testing group of the remaining 30%, with the testing group being larger than general machine learning tests to ensure accurate representations of the evaluation metrics with a limited dataset. Each of the four regression models was then fit to the training data and asked to predict on the testing set. Finally, for a visual representation, predicted vs. actual arsenic concentration was graphed for each model.

#### 2.4.2. Dimensionality Reduction with Genetic Algorithm

Due to the large volume of data hyperspectral remote sensing offers, literature suggests that, without the extraction and use of characteristic wavelengths, data redundancy reduces the accuracy of any relationship-establishing regression model [21]. In addition, due to the limited dataset of 55 output locations available for this study, the results of using 155 input wavelengths might be somewhat untrustworthy; using a characteristic wavelength-derived dataset can help ensure greater validity of this study’s results.

For this study, characteristic wavelengths were extracted using the highest weights generated by the Genetic Algorithm (GA). GA is an optimization algorithm based on Darwinian evolutionary principles of survival-of-the-fittest [45] that iterates on a randomized given population until it selects the most relevant variables, all while considering the possible interactions between these variables [18]. Here, the variables are the input hyperspectral wavelengths and their values, so the result is the most important (“characteristic”) wavelengths for predictions.

GA was coded with a population size of 98, 155 weights, and 1000 generations. The characteristic wavelengths resulting from GA were combined with the best performing regression model and evaluated in the same way as other regression models (comparisons of $R^{2}$ and nRMSE). The resulting characteristic wavelengths were also compared with previously identified As characteristic wavelengths as well as those of other minerals found in literature to be correlated with spectroscopic arsenic identification such as Fe-oxides, organic matter content, and clay [46].

#### 2.4.3. Data Augmentation

It is well-known that machine learning models tend to overfit and be unreliable on smaller data sets. To attempt to overcome this, data augmentation with The Synthetic Minority Oversampling Technique for Regression with Gaussian Noise (SMOGN) was used. SMOGN is particularly effective with imbalanced regression datasets such as the one in this study [47]. The algorithm works by finding distances between a point and its k-nearest neighbors; if the distance is less than or equal to half the median of all distances, then interpolation is used [48]. Otherwise, Gaussian noise, statistical noise having a probability density function equal to the normal distribution, is introduced [47]. With SMOGN, the dataset of 55 points was expanded to 98 points, with the expanded dataset being used to generate a new model based on the best-performing one from earlier. The new model’s $R^{2}$ and nRMSE values and scatter plot were compared to earlier models’ results.

### 2.5. Classification Analysis with High-Risk Shrub Soil Regions

A classification-based approach was applied to detect arsenic risk in shrub-covered soils in order to broaden the applicability of the methodology to larger areas. A similar approach with bare soil could not be tested due to limited bare soil data availability. While such a classification approach is less precise than regression, only being able to distinguish the highest-risk regions instead of accurately predict specific numerical values, it offers a greater advantage in extending applicability to a more common land cover and for larger regions.

Binary machine learning classification models were applied to identify higher-risk regions of the United States based on risk of arsenic contamination, with the threshold for a “high-risk” region chosen to be 7.0 mg/kg, the 70th percentile cutoff for arsenic risk classes. Large-scale risk classification was done for random locations with shrub land data (obtained from publicly available USGS data [49]) from ten U.S. states: Arizona, California, Colorado, Idaho, Nevada, New Mexico, Oregon, Texas, Utah, and Wyoming. A dataset of 20,000 locations was determined by randomly picking points from 11 swaths. Approximately 30% of the dataset consisted of high-risk locations, as consistent with the cut-off of high-risk arsenic concentrations used in this study (the 70th percentile), with all percentiles attempted to be as equally represented as possible with the available data. An 80/20 train–test split of the dataset was initially used for prediction testing. After initial failure of the simple 80/20 train–test split on the whole dataset in sufficiently evaluating classification results (to be discussed in the Results and Discussion section), for more robust analysis, each model was run 11 times, with each run using a different swath’s data as the testing set and the other 10 of the 11 swaths as training dataset. Each evaluation metric was then averaged across these 11 different results to yield the final comparison metrics for the three models. Other dataset modifications were also attempted, as described later in the Results and Discussion section.

Three relevant binary machine learning classification models were tested: Support Vector Machine (SVM), Random Forest Classification (RFC), and Multi-Layer Perceptron (MLP). SVMs are the most commonly used classification models in previous studies of hyperspectral data, working well in datasets with high dimensionality but not as suitable for larger and/or noisier data sets [50]. RFC boasts systematic handling of high-dimensionality datasets and good adaptability with imbalanced datasets [51]. Finally, the MLP deep learning classification model has advantages in generalizing trends and updating errors effectively, similar to BPNNs. GA and SD were used on the dataset with each of the three tested classification models as base preprocessing in order to maximize computational efficiency and performance. All three models were optimized (Table 1).

Models were evaluated and compared based on five metrics:

1. Accuracy: The percentage of the total number of classification predictions that are correct. A perfect classifier has an accuracy of 1.0.
2. F1-Score: A score combining precision—how many high-risk points predicted to be high-risk are actually high-risk—and recall—how well the high-risk points were predicted overall. A perfect classifier has a F1-score of 1.0.
3. F2-Score: A score similar to the F1-score but with more of a focus on recall calculations to measure presence of false negatives—actual high-risk regions predicted as low-risk.
4. F0.5-Score: A score similar to the F1-score but with more of a focus on precision calculations to measure presence of false positives—actual low-risk regions predicted as high-risk.
5. Brier Score: A score that measures the uncertainty of a model’s probabilistic predictions, prioritizing the high-risk regions. A perfect classifier has a Brier score of 0.0.

## 3. Results and Discussion

### 3.1. Land Cover Verification

**Figure 4:**
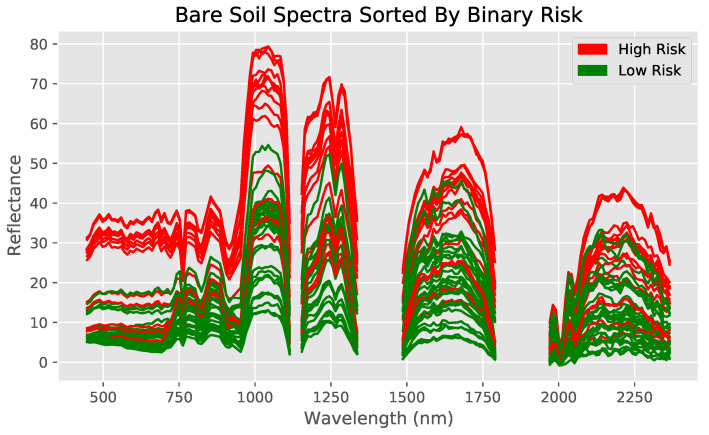
Hyperspectral data measured by EO-1 Hyperion, with bare soil spectra sorted into low and high risk regions. Spectra for each of the two classes visually differ clearly across the whole spectrum of wavelengths between 400 nm and 2400 nm. High risk is defined as the 70th percentile (7.0 mg/kg) and above.

**Figure 5:**
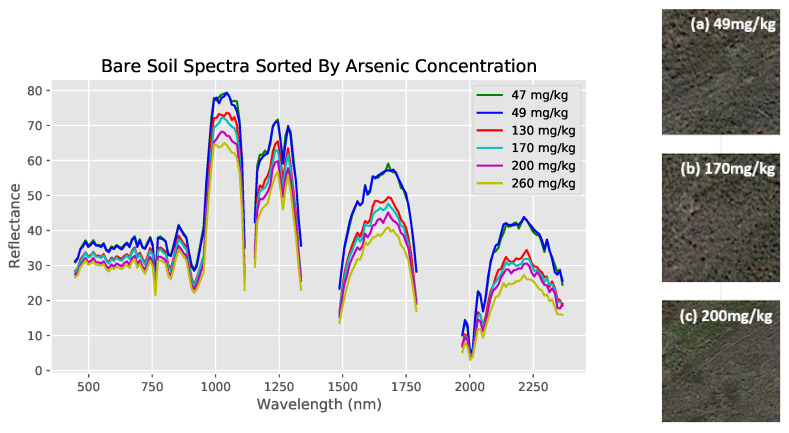
Comparing reflectance spectra of six bare soil locations with differing arsenic concentrations from ground measurements. Sites have similar soil composition and visual satellite appearance, as shown on the right.

**Figure 6:**
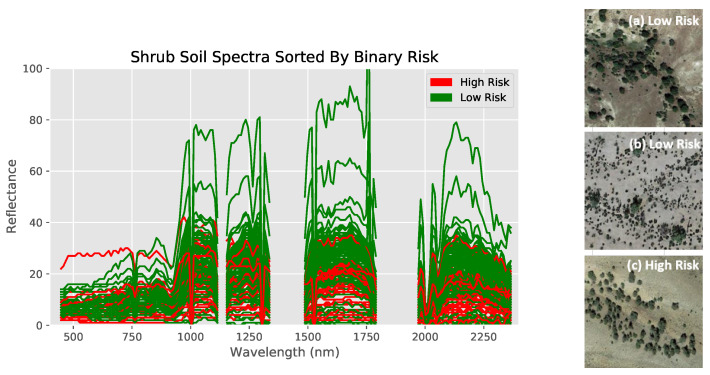
Comparing reflectance spectra of locations with differing arsenic concentrations but similar soil composition and visual satellite appearance for shrubland. Sites have similar soil risk and visual satellite appearance, as shown on the right. Overall, some differences can be seen, but they are not as visibly distinct as with bare soil.

Bare land cover comparisons were done with numerical data from the regression dataset (Figure 4 and Figure 5). Note that the empty regions of the spectra result from wavelength bands removed during preprocessing. Furthermore, observe how similar the 47 mg/kg and 49 mg/kg spectra are to each other (indicating the necessity of the use of machine learning models to identify specific differences), while both are clearly different from their higher concentration counterparts (Figure 5). Shrub land cover comparisons originated from the classification dataset, with spectra from previously defined high and low risks used instead (Figure 6).

The spectra of both land covers show at least some clear visual differences between different arsenic concentrations despite otherwise similar conditions, indicating that the methodology likely highlights arsenic concentration and not land cover. Furthermore, visual differences between specific numerical values can be recognized in the bare soil regression dataset, while this is not the case for the shrubland dataset (hence requiring a binary classification approach for shrub-covered soils).

### 3.2. Noise Reduction Algorithms (Spectral Preprocessing)

**Figure 7:**
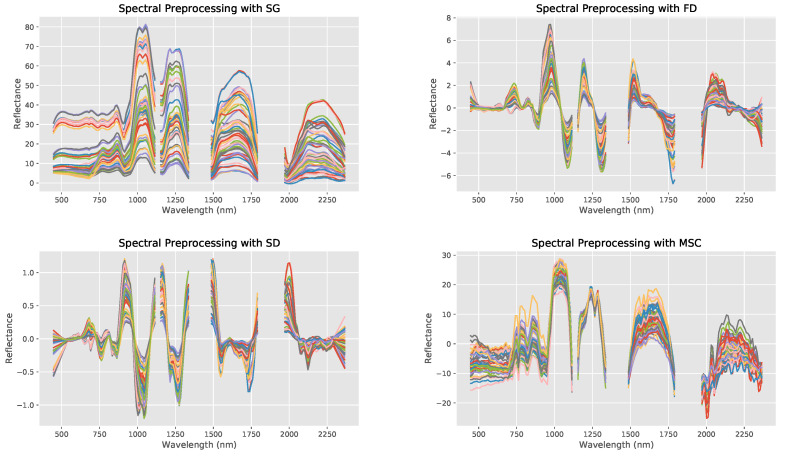
The spectral reflectance curves of the bare-soil arsenic samples after spectral preprocessing through: SG (**top left**), FD (**top right**), SD (**bottom left**), and MSC (**bottom right**). Line colors are used to differentiate samples only.

First, the original spectrum, SG, FD, SD, and MSC spectral preprocessing were visually compared with each other to see the effects of each kind of spectral preprocessing (Figure 7).

Out of the five, the SD transformation had the highest $R^{2}$ value of 0.623 when combined with the PLSR machine learning model with an nRMSE value of 0.194. After SD transformation, MSC performed best in terms of $R^{2}$ value, followed by FD, SG, and finally the unprocessed spectrum (Table 2). SD transformation also yielded the lowest nRMSE values besides that of the FD transformation, which was actually quite a bit lower than FD’s. In the future, further analysis needs to be performed about the trade-offs between improved $R^{2}$ values but decreased nRMSE values, but for the purposes of this study, $R^{2}$ was given higher priority in model evaluation than nRMSE as the study focuses on the best possible correlation determination.

Noise reduction with SD transformation is the most effective likely because it can highlight otherwise hard to discern absorption features of spectral curves [52]. Furthermore, taking two derivatives allows for the clearer differences to be observed between spectral reflectance of indicative bands with different arsenic contents, which served to increase model accuracies more than the other kinds of transformations. Therefore, SD transformation was chosen to be paired with all following models to maximize predictive capabilities.

### 3.3. GA-Generated Characteristic Wavelengths

Running the Genetic Algorithm (GA) generated a set of weights for each of the 155 used wavelengths. The top ten best-performing wavelengths (highest weighted) were selected as the characteristic wavelengths (in nm): 495, 650, 885, 1225, 1568, 1679, 2143, 2183, 2203, and 2254. These 10 chosen characteristic wavelengths are fairly consistent with characteristic arsenic wavelengths reported in previous studies [26,53].

They also align well with soil Fe-oxide content. Fe-oxides in soil are generally present as two main minerals: goethite and hematite. Goethite has its main peak around 920 nm, with four supplementary ones at 420, 480, 600, and 1700 nm; hematite has three main peaks at 520, 650, and 880 nm [54]. While not perfect, the GA-chosen ten characteristic wavelengths contain wavelengths fairly close to all of these peaks besides goethite’s 420 nm supplementary peak (a wavelength filtered out in spectral preprocessing due to atmospheric influences, something which previous lab-based analyses of soil reflectance spectra do not need to take into account). The correlation is especially strong with hematite.

Most clay minerals have diagnostic wavelengths of approximately 1400, 1900, and 2200 nm, representing the influences of OH, water, and Al-OH groups, respectively [54]. Wavelengths near and including the first two wavelengths (1400 nm and 1900 nm) had to be filtered out due to atmospheric influences, but we do see that four out of ten characteristic wavelengths lie near the 2200 nm diagnostic, which suggests that there might be some correlation with clay that unfortunately cannot be confirmed with satellite data.

Finally, little correlation was found with organic matter based on the characteristic wavelengths [55].

### 3.4. Regression Models with Bare Soil Regions

#### 3.4.1. Testing ML Regression Models Using 155 Wavelengths

**Figure 8:**
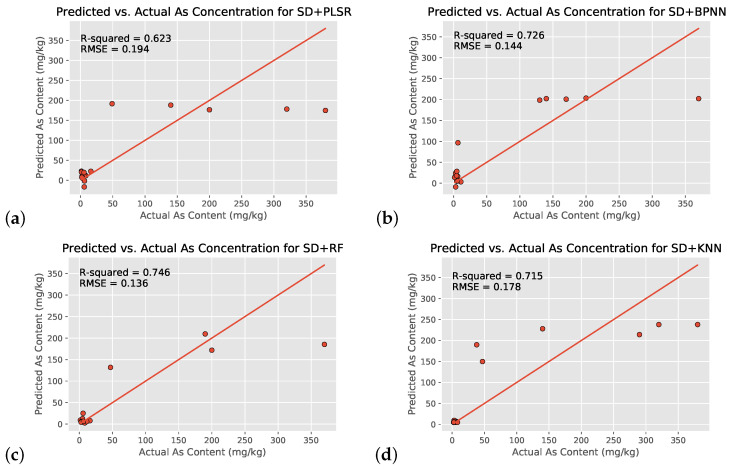
Scatter plots of the soil arsenic concentrations after the use of four different machine learning models with all 155 input wavelengths and SD transformation (SD+Reg models): PLSR (control group, (**a**)), BPNN (**b**), RF (**c**), and KNN (**d**).

With the use of all 155 wavelengths on the 55-location dataset, the SD+RF regression model yielded the highest $R^{2}$ value of 0.746, followed by SD + BPNN, SD + KNN, and SD + PLSR (Figure 8a–d and Table 3). All four machine learning models showed moderately strong correlation between preprocessed hyperspectral satellite imagery and environmental arsenic contamination, likely indicating that such a relationship does indeed exist and is not a coincidence. All three nonlinear models also exceeded the linear PLSR, indicating that the relationship between the two variables is indeed nonlinear as consistent with literature. Furthermore, overfitting did not occur in the SD + RF model, as the training and testing set $R^{2}$ values were within 0.05 of each other ($R^{2}=0.788$ vs. $R^{2}=0.746$).

#### 3.4.2. Testing ML Regression Models Using GA-Generated Characteristic 10 Wavelengths

**Figure 9:**
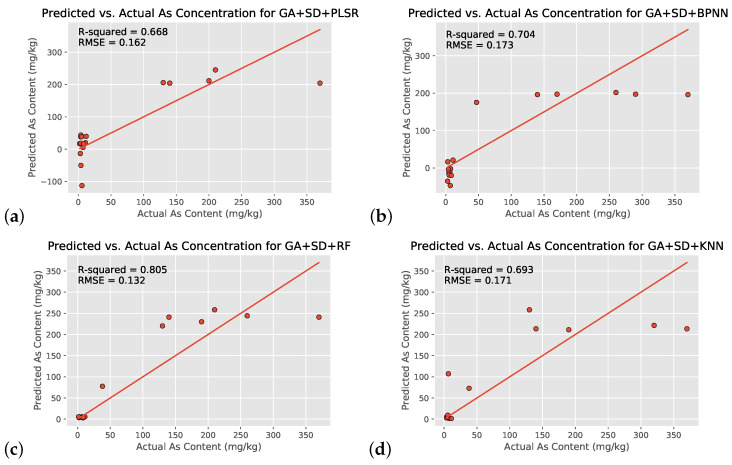
Same as Figure 8 but with GA-Generated 10 input wavelengths and SD transformation (GA + SD + Reg models): PLSR (control group, (**a**)), BPNN (**b**), RF (**c**), and KNN (**d**).

Because of the small regression dataset size (55 output arsenic concentrations vs. 155 input wavelengths), the characteristic 10 wavelengths generated by the use of the Genetic Algorithm were used as a new dataset to validate the original regression’s results (Table 4 and Figure 9). Narrowing down the dataset to the characteristic wavelengths chosen by GA still maintained the RF regression model as the best one, with the GA + SD + RF model showing an increase of 0.059 ($R^{2}=0.805)$ in $R^{2}$ value and decreases in nRMSE value and computational time. The linear PLSR model showed comparable improvements.

In contrast, the BPNN and KNN models both performed worse with the characteristic wavelengths, having decreases in their $R^{2}$ and/or nRMSE values. The probable reason for this is that some of the 155 wavelengths that were removed were actually important to informing the results of these two models, with these wavelengths likely much less important to the PLSR and RF models.

While this study’s regression models are a good overall fit for the data, most graphs in Figure 8 and Figure 9 have data points noticeably far from the 1:1 line of equivalence. In order to include a larger range of arsenic concentrations to ensure greater applicability, the dataset was chosen to include values ranging from 1.4 mg/kg to 380 mg/kg. This leads to the clustering of data at very small values on the graph, as more than 70% of the included data points are between 1 and 10 mg/kg, and the remaining ones are concentrated on the higher end of the range with few in the middle. Furthermore, graphs (a)–(d) in Figure 8 and Figure 9 indicate that all four models can clearly distinguish between the lower-risk and higher-risk arsenic concentrations (as consistent with Figure 4) but might not be able to identify a consistent trend in the higher-risk values (likely because of a shortage of training data). In addition, despite thorough preprocessing, the data might still be affected by atmospheric interference, instrument issues, etc., which could be affecting correlation. In order to improve the estimation accuracy of soil arsenic content, it is necessary to collect more verified soil samples to build a more even distribution of concentrations in the future.

Despite BPNN’s noted past excellency working with hyperspectral data and deriving correlations [21,22], it performed worse than RF. While the difference is small, BPNNs are known to be prone to a lack of generalization ability and hence are likely not able to fully optimize their initial parameters. Meanwhile, RF regression is known to be particularly good with high dimensionality datasets such as the one in this study and effectively handles outliers by “binning” them. It was likely a combination of these factors that led to the better performance of the SD+RF model. The KNN regression model performed slightly worse than the BPNN, probably because of its sensitivity to outliers.

#### 3.4.3. Applying Data Augmentation

**Figure 10:**
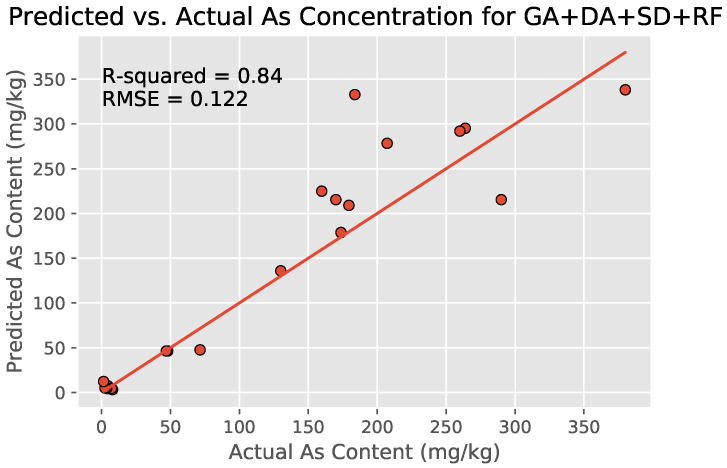
Scatter plot of the soil arsenic concentrations after the use of the DA + GA + SD + RF model with DA-increased dataset, GA-Generated 10 input wavelengths, and SD transformation. The model outperforms all other combinations tested in this study ($R^{2}=0.840$ and nRMSE =0.122).

SMOGN-based DA (expanding the number of locations from 55 to 98 locations) combined with the earlier best-performing GA + SD + RF model yielded a higher overall $R^{2}$ value of 0.840 for the new DA + GA + SD+RF model with nRMSE =0.122 (Figure 10). The nRMSE of the model decreased with lesser magnitude (0.01) as compared to the increase in $R^{2}$ value (0.035), possibly because of the addition of Gaussian noise.

The finalized DA + GA + SD + RF regression model was also comparable to ones from literature, especially considering that the others had access to more accurate and less noisy lab-based hyperspectral measurements (Table 5).

### 3.5. Binary Risk Classification for Shrub Regions

A dataset of 20,000 locations from ten different U.S. states was used, and three binary classification models—SVM, RFC, and MLP—were tested for identifying high-risk arsenic regions. Initially, the model was tested with all 20,000 points at once, randomly choosing 20% of the data for testing purposes. This approach yielded an extremely impressive accuracy of 0.932 by the MLP model, which seemed unrealistic given previous regression results. Running the models 11 times, with each run using a different whole swath’s data as the testing dataset and the other 10 of the 11 swaths as training data, would help test the hypothesis that machine learning models were only learning by geographical similarity as opposed to establishing a legitimate correlation; although each swath has very similar hyperspectral values (spatial homogeneity), the swaths used were all from different states, and testing one swath at a time with training data consisting of the other 10 swaths would likely force the model to attempt to determine a real correlation instead of “learning by location”. With this new approach, the MLP model still performed best across four of the five tested metrics (all but the Brier score) (Table 6), still achieving a strong (and much more realistic) averaged accuracy of 0.693. The other models were also effective in binary classification, though inferior to the MLP. This was likely because of MLP’s powerful iteration through errors, something that is especially important in classification problems with large datasets.

This study deals with an imbalanced training dataset of 70% low-risk locations and only 30% high-risk ones, so accuracy is not necessarily the most effective metric to determine model performance. As an example, a no-skill model could theoretically guess that all points are low-risk ones and get approximately 70% accuracy that way as well. Although this was not the case here as evident from individual swath results, it was why four additional metrics were evaluated—F1-score, F2-score, F0.5-score, and Brier score.

As the ratio of high-risk to low-risk points present in the dataset matches the real-world arsenic risk percentiles, and identification of the high-risk class (“positive class”) is more important than its low-risk counterpart, the three types of F-scores serve as excellent evaluation metrics. The best-performing MLP model achieves an F1-score of 0.728, 0.04, and 0.05 points higher than SVMs and RFCs, respectively, and indicative of good model performance [57]. However, while it is important to identify false positive predictions because they can generate false alarms that could lead to wastage of resources, arguably, not correctly identifying a high-risk region (false negatives) might be much more costly to human lives and resources because of healthcare, cleanup, and other associated economical costs. Fortunately, the MLP model’s results alleviate these concerns given its strong F2-score (=0.704) and F0.5-score (=0.772), which are each better than the other tested models. Put another way, the MLP model, given a location to predict risk of, is 22.8% likely to output a false warning of the location being high-risk and 29.6% likely to misidentify a high-risk region as a low-risk one. These results further indicate that the MLP model is less likely to generate false warnings of arsenic risk and misidentify risky regions as compared to traditional field-based monitoring approaches (which generated inaccurate results almost 45% of the time [14]).

The probability-based Brier score metric, with its priority on the positive class, helps measure the reliability of a model, not only measuring when it fails but also whether it selects the wrong class with a high or low probability [57]. The otherwise best-performing MLP, perhaps surprisingly, performed the worst in terms of Brier scores, achieving 0.279 as opposed to SVM’s 0.272 and RFC’s 0.252 (a perfect classifier should display a Brier score of 0.0). This indicates that, when the binary classification models are asked to describe the probability that a certain region is high-risk (a more continuous prediction in the range [0,1]) instead of thinking about it in a purely binary fashion, the MLP model performs slightly worse than the other two tested models. In other words, the MLP model is more unsure about its predictions, which is interesting to take into account and its implications can be further analyzed in future studies when compared to its superior performances in accuracy and the various F-scores.

**Figure 11:**
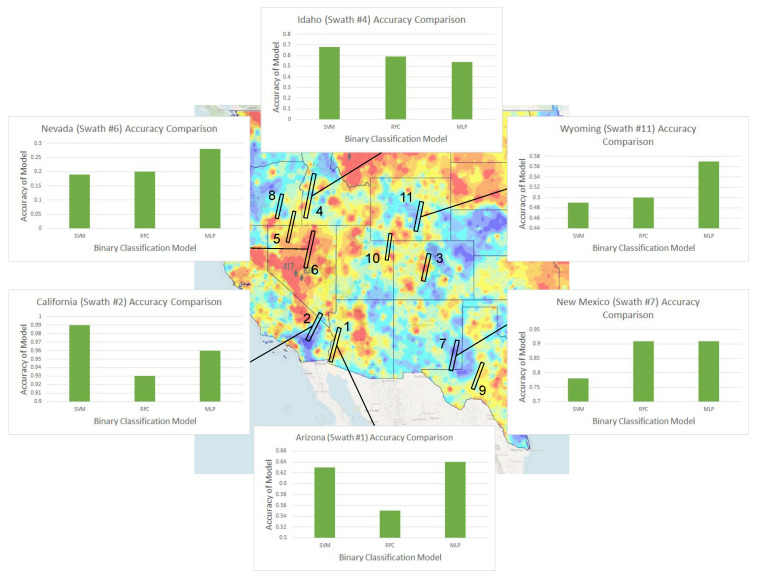
Comparison of accuracy of three binary classification machine learning methods for six sample swaths. Background map of the western US colored by arsenic risk [49] showing the chosen 11 swaths (which yield 20,000 points) for shrub soil arsenic risk analysis (swath signatures are presented in Table 7 for public reference, being obtained from the USGS Earth Explorer platform). The results of the three binary classification machine learning models are shown as bar graphs for 6 of the 11 swaths. Accuracy values for all swaths are shown in Figure 12. Background mage created with USGS data [49].

**Figure 12:**
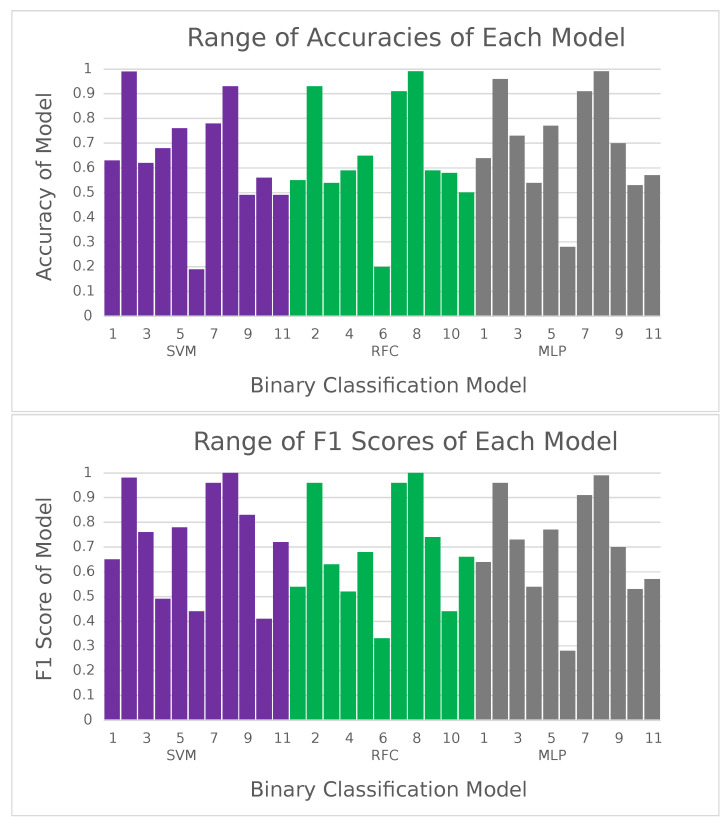
Bar graphs showing the accuracies (**top**) and F1-scores (**bottom**), in alphabetical order, of each of the three models for each of the chosen 11 swaths for shrub soil arsenic risk analysis. Numbers below bars correspond to swaths in Figure 11.

Besides the averaged metrics, a few of the individual results of the 11 swaths (Figure 11 and Figure 12) warrant additional discussion.

The Nevada Swath (#6) yields the worst scores out of all 11 swaths for all five metrics examined, with even the best-performing MLP model only displaying 28% accuracy and an F1-score of 0.44. This is also the only swath comprising of only high-risk locations (2000 of them), which probably explains its poor performance—as there are only 6200 total high-risk points in the dataset, taking out almost a third of them while training likely leads to insufficient coverage of adequate training data; similarly, it is also possible that the data from Nevada adds something significantly new to the training data that the binary classification models use when informing their predictions, and thus its absence leads to poor model results on its testing.

In contrast, the Oregon, California, and New Mexico swaths show abnormally high evaluations across all five metrics (accuracies and F1-scores greater than 0.9 for each of the three), sometimes up to almost perfect performance with specific ML models. This possibly indicates that enough relevant information probably exists in the training dataset without the presence of just one of these swaths. Another possible reason could be that the geography (and hence reflectance) in these regions is quite similar, which allows for the model to infer the tested swath’s predictions based on the other two in the training dataset (similar to the idea of spatial homogeneity discussed earlier that led to the 93% accuracy). Perhaps interestingly, all three swaths only have low-risk points.

To compensate for some of the poor performance of high-risk predictions such as with the Nevada swath, 5000 additional high-risk locations were added to the dataset from Nevada, Arizona, and Colorado, leading to 45% of the 25,000-point dataset now comprising of high-risk regions (an almost balanced dataset) for training and testing. However, especially taking into account that moving towards a more balanced dataset should skew the values of the resulting F-scores higher, the results of these additions yielded almost negligible benefits in terms of all five metrics. Each previously affected swath also did not perform more than a few tenths of a percent better than the original MLP model with 20,000 points. These results indicate that adding just a small number of additional points yields diminishing returns in terms of model output and prediction reliability, and is not effective since the current dataset already spans the targeted region (the Western United States) fairly well. While adding in a much larger number of both low-risk and high-risk points (for example, making the dataset 200,000 points or larger—a 10-fold increase in dataset size) might give further insights, small additions only serve to worsen computational efficiency without offering major improvements.

### 3.6. Limiting Factors of the Satellite Approach

The use of satellites involves many complications that must be considered in order to appropriately evaluate its applicability for large-scale arsenic contamination detection. The most important of these challenges include the time of day of measurements and solar inclination angle, relief characteristics, extent and type of vegetation, and atmospheric obstacles such as humidity and cloud cover. Anticipating this, NASA included many ground truth verification locations in Hyperion’s initial paths to ensure effective calibration [58,59]

Because of the presence of visible light wavelengths in the examined spectra, the time of day of satellite measurement is certainly a restrictive condition in terms of method applicability. Future analysis should be conducted to test the effectiveness of only using infrared wavelengths to detect arsenic contamination; if the resulting accuracies are reasonably similar to current ones, the impact of time of day can be greatly reduced because of infrared’s relative independence from time of day.

Another potential solar limitation is the solar inclination angle, the angle of the orbit in relation to Earth’s equator. NASA data contain solar inclination angles between 20 and 90 degrees, with mostly smaller angles (25–40 degrees) between 2001 and 2009 and larger angles in later years such as 2015 [60]. All possible solar inclination angles, fortunately, can be corrected for by the formula presented in Section 2.2. Most other sun-related satellite issues are corrected for by NASA’s solar calibration of the Hyperion satellite [61], thus likely only contributing minor deviations.

Throughout its orbit, the Hyperion satellite maintained an orbital inclination between 98 and 99 degrees. This indicates that its measurements of swaths were done almost perpendicularly to ground level, which minimizes potential topographical distortions [62].

Most locations used in this study are in relatively flat areas, so topographic variations are not a concern. In addition, NASA’s base-level geometric calibration is able to address small changes in relief. One study found that slopes greater than 15 degrees introduced a peak value in anomaly trends, with minimal disturbances in angles less than 15 degrees [60]. Therefore, significantly sloping topography could certainly be a limitation on the accuracy of this study’s proposed methodology due to difficulties in geometric correction and the higher potential for arsenic mobility. Applying the methodology to such a region might be limited to binary risk analysis, and additional ground truth verification would be required.

Some of the effects of the extent and type of vegetation have already been discussed in detail in Section 2.2 and Section 3.1. Any kind of ground cover with extensive soil coverage—forests, crop fields, and densely-covered grass and shrub soils—would be unsuitable for a satellite approach. However, many regions containing these types of cover have particular times of the year when the extent of soil coverage is reduced. For example, most crop fields globally only have certain months of planting and harvest, with other months exposing relatively bare soil. Although it would require additional coordination and resources, hyperspectral measurements could be scheduled accordingly in order to allow for accurate annual measurements in these regions.

Finally, atmospheric hindrances must be considered. Before inclusion, every swath used in this study was visually examined to ensure minimal cloud cover. In the future, an image-based machine learning algorithm could be developed to automate this process. Most “bad bands” of the Hyperion satellite, including those containing effects of water vapor (humidity), were systematically preprocessed and removed, as described in Section 2.2. The use of characteristic wavelengths further filtered out problematic, redundant wavelengths. As a result, while atmospheric challenges might still be present, most major consequences were sufficiently addressed and can also be feasibly addressed in future studies. While a paid professional software such as ENVI’s FLAASH might be better suited to more carefully and accurately eliminate atmospheric disturbances, the method used here offers a close approximation for no monetary cost and faster computational times. Future studies might look at how results derived from using such software compare with the results of this study.

Overall, the aforementioned factors can play large roles in limiting the applicability of a satellite-based approach in certain regions of the world, particularly those with frequent climatic fluctuations, successive topographical changes, and year-long soil cover. However, outside of these regions, their effects on the accuracy and applicability of the presented methodology are adequately addressed using thorough preprocessing.

### 3.7. Additional Future Directions

Due to lack of publicly available data, this study’s models cannot distinguish between different forms of arsenic such as trivalent (arsenite) and pentavalent (arsenate) arsenic. Inorganic arsenic variants vary in their toxicity, which could lead to false alarms without proper identification. Being able to distinguish between different forms of inorganic arsenic is an important future goal to be able to accurately determine the scale of impact and spread of arsenic contamination. One potential approach to classify arsenic into its various forms would be to measure the differences in lab-obtained spectra with differing concentrations/ratios of these components, similar to how the reflectance spectrum of overall arsenic varies with different concentrations. A similar approach might be possible to help distinguish between natural and anthropogenic forms of arsenic in the environment. Based on the results of this study, it is probable that there are detectable differences between arsenic content originating from two different chemical sources.

Arsenic is frequently heard of in the context of groundwater contamination, which is difficult to approach directly with satellites as the water table often lies below the surface and is not easily detectable through direct hyperspectral measurement. However, literature has shown that most of the increased arsenic content in groundwater does originate from soil-based sources and activities such as mining activity, fossil fuel combustion, and pesticide runoff [63]. One study found a correlation as high as 92.9%, with p-value less than 0.0001 [64]. The study concludes that there is a possibility of at least an above average correlation between soil and groundwater arsenic, implying that this study’s methods might also be applicable to predicting groundwater arsenic contamination, though with less certainty.

An important future goal of this study is to apply the predictive power of the DA + GA + SD + RF and MLP models to arsenic risk zones in developing countries, which often have poorer sampling and response measures to environmental contamination hazards such as arsenic. The models are expected to perform with good accuracy, and success in this endeavor would likely establish the models as being capable of feasible implementation by universities, nonprofits, and governments globally in the future.

Overall, this study’s strong regression and especially classification results serve as a valid proof-of-concept for a similar methodology to be applied more broadly in the real world, but more work needs to be done before viable implementation. Future studies can aim to improve metric scores to achieve better classification, possible through more robust preprocessing or the inclusion of many more data points; test other land covers such as grasslands and crop fields; introduce more classes into the dataset (for example, five categories of risk as opposed to binary classification) in order to obtain more precise risk analyses for vulnerable regions; test other machine and deep learning models, preprocessing algorithms, optimization techniques, or some other novel combination of the three; and apply the adaptable nature of this study’s methodology towards detection of other types of environmental contaminants such as lead and cadmium.

## 4. Conclusions

In the first part of this study, regression-based machine learning models were used to determine a correlation between a location’s hyperspectral satellite wavelength data and bare soil arsenic concentration. With its contiguous spectrum and high spectral resolution, hyperspectral satellite data from NASA’s EO-1 Hyperion were hypothesized to allow for the distinguishing of small differences in soil arsenic concentrations of different regions. The second part of this study involved the use of binary classification-based machine learning models to identify high-risk shrub-covered soil zones in ten U.S. States. The following are the most important conclusions derived from this study:

1. SG, FD, SD, and MSC transformations were performed on the spectral data, and a regression model was established by PLSR. The SD transformation had the best modeling accuracy ($R^{2}=0.623$), a 0.131 increase over no preprocessing.
2. All three nonlinear machine learning regression models outperformed the linear model, validating the hypothesis that the relationship between the hyperspectral wavelengths and soil arsenic concentration is nonlinear. SD + RF was the most effective out of the four tested models in determining correlation ($R^{2}=0.746$ and nRMSE = 0.136).
3. GA is effective in selecting characteristic wavelengths that align well with previously indicated characteristic wavelengths for arsenic as well as Fe-oxides and clay. The GA + SD + RF model, using only these wavelengths, yielded improved model accuracy ($R^{2}=0.805$), nRMSE (=0.132), and computational efficiency.
4. Data augmentation with SMOGN increases regression dataset size by 78%, and the final regression model (DA + GA + SD + RF) achieved $R^{2}=0.840$, nRMSE = 0.122, and good computational efficiency.
5. The MLP binary classification model is effective in identifying high-risk arsenic regions in shrub-covered soils using Hyperion satellite imagery (Accuracy = 0.693, F1-Score = 0.728, F2-score = 0.704), with much lower false alarm and incorrect identification rates as compared to traditional methods. Both conditions tested (shrub regions and classification) are novel applications made possible because of the large-scale and efficient use of satellites over lab-based instruments.

These results strongly indicate that soil arsenic contamination can be detected with Hyperion satellite hyperspectral data when combined with preprocessing and machine learning. At the very least, this study’s models can be used as a screening tool to identify high-risk regions in applicable land covers, reducing the need for frequent sampling of large-scale regions. Overall, the results of this study open up possibilities for a cost-effective, rapid, and versatile annual monitoring system of environmental arsenic contamination.

## Figures and Tables

**Figure 3:**
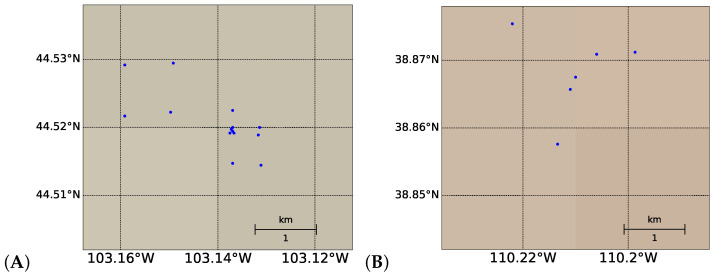

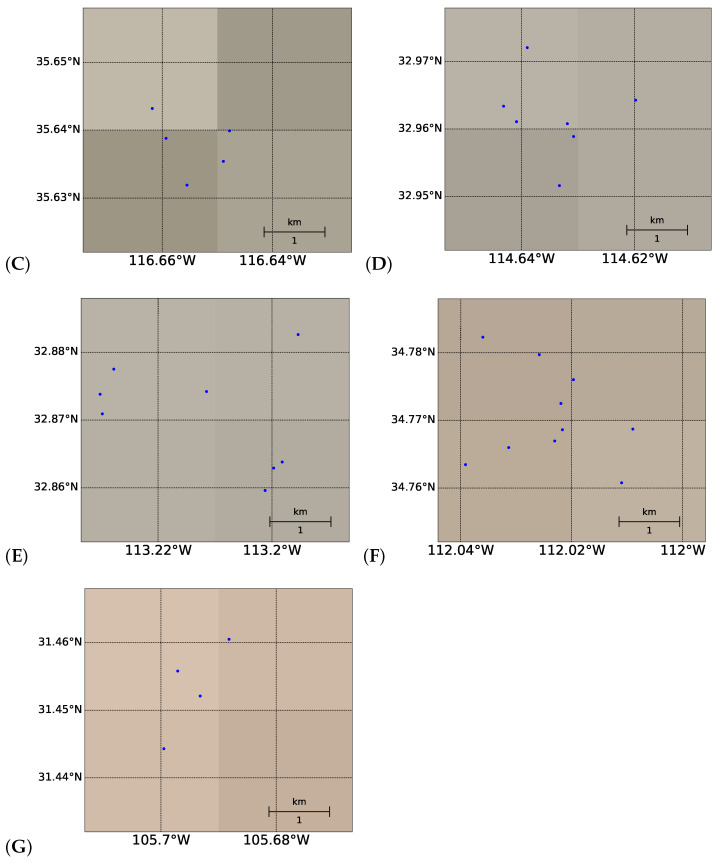
Data from USGS sites in sub-regions (**A**–**G**), with each specific location in each sub-region shown in subplots 2 (**A**–**G**), as labeled in Figure 2. Differences in background color are topographic variations (not easily visible due to the zoom-in on the relevant regions).

**Table 1:** Optimized regression (Reg) and classification (Cls) machine learning algorithm parameters in Python scikit-learn. If a parameter is not explicitly mentioned, it was left to default Scikit-learn definitions. Refer to the Abbreviations section at the end of the paper for full model names.

ML Model Tested	Parameters
PLSR Reg	n_components = 4, max_iter = 40
BPNN Reg	See text in Section 2.4.1, not done with Scikit-learn
RF Reg	n_estimators = 30, random_state = 0
KNN Reg	n_neighbors = 4, weights = ‘distance’
SVM Cls	C = 10, kernel = ‘rbf’, probability = True
RFC Cls	n_estimators = 200, criterion = ‘gini’, random_state = 0
MLP Cls	activation = ‘tanh’, solver = ‘adam’, learning_rate = ‘adaptive’,
	early_stopping = True

**Table 2:** Comparison of the $R^{2}$ and nRMSE values of the four noise reduction algorithms tested. Refer to the Abbreviations section at the end of the paper for full technique names.

Algorithm	$R^{2}$	Normalized RMSE
None (unprocessed)	0.492	0.227
Savitzky-Golay Smoothing (SG)	0.546	0.203
First Derivative Transformation (FD)	0.557	0.148
Second Derivative Transformation (SD)	0.623	0.194
Multiplicative Scatter Correlation (MSC)	0.565	0.215

**Table 3:** Table of the comparison of the $R^{2}$ and nRMSE values of the four regression-based machine learning algorithms tested for bare soil arsenic detection using all 155 wavelengths. Refer to the Abbreviations section at the end of the paper for full technique names.

Algorithm	$R^{2}$	Normalized RMSE
SD + Partial Least Squares Regression (PLSR)	0.623	0.194
SD + Back Propagation Neural Network (BPNN)	0.726	0.144
SD + Random Forest Regression (RF)	0.746	0.136
SD + K-Nearest Neighbors (KNN)	0.715	0.178

**Table 4:** Table of the comparison of the $R^{2}$ and nRMSE values of the four regression-based machine learning algorithms tested for bare soil arsenic detection using the GA-Generated 10 characteristic wavelengths. Refer to the abbreviations section at the end of the paper for full technique names.

Algorithm	$R^{2}$	Normalized RMSE
GA + SD + Partial Least Squares Regression (PLSR)	0.668	0.162
GA + SD + Back Propagation Neural Network (BPNN)	0.704	0.173
GA + SD + Random Forest Regression (RF)	0.805	0.132
GA + SD + K-Nearest Neighbors (KNN)	0.693	0.171

**Table 5:** Comparison of previous lab-based estimation models of arsenic concentration in bare soils with the satellite-based best-performing DA + GA + SD + RF model from this study.

Sample Size	Model Methods	$R^{2}$	References
93	SG + PLSR	0.750	[53]
61	FD + PLSR	0.720	[56]
96	GA + PLSR	0.35	[20]
89	sCARS-SPA + SFLA + RBFNN	0.918	[21]
90	PSO + BPNN	0.811	[22]
98	DA + GA + SD + RF	0.840	This study

**Table 6:** Comparisons of the evaluation metrics of the three binary classification ML models for the averaged swath data.

Model	Accuracy	F1-Score	F2-Score	F0.5-Score	Brier Score
SVM	0.647	0.688	0.658	0.751	0.272
RFC	0.639	0.678	0.649	0.737	0.252
MLP	0.693	0.728	0.704	0.772	0.279

**Table 7:** NASA-designated signatures, along with the U.S. state, of the 11 swaths used in shrubland arsenic classification.

Swath #	U.S. State	Signature
1	Arizona	EO1H0380362012066110PB
2	California	EO1H0390352017062110KF
3	Colorado	EO1H0340332003257110PY
4	Idaho	EO1H0410292007009110PZ
5	Neavda & Oregon	EO1H0420312011146110P0
6	Nevada	EO1H0410322005112110KX
7	New Mexico	EO1H0310382003092110PZ
8	Oregon	EO1H0430302003144110PZ
9	Texas	EO1H0300382008351110PY
10	Utah	EO1H0360322003177110KW
11	Wyoming	EO1H0360302003278110KZ

## Data Availability

Only publicly available data sets were analyzed in this study. Data for arsenic concentrations can be found at https://www.waterqualitydata.us/ and https://mrdata.usgs.gov/ds-801/ (accessed on 13 September 2021). Hyperspectral satellite data was obtained from NASA’s EO-1 Hyperion satellite, and the data can be found at https://earthexplorer.usgs.gov/ (accessed on 13 September 2021). Specific resulting data sets presented in this study are available on request from the corresponding author.

## Abbreviations

The following abbreviations are used in this manuscript:

SG	Savitzky–Golay Smoothing
FD	First-Derivative Transformation
SD	Second-Derivative Transformation
MSC	Multiplicative Scatter Correlation
PLSR	Partial Least Squares Regression
BPNN	Back Propagation Neural Network
RF	Random Forest Regression
KNN	K-Nearest Neighbors
$R^{2}$	coefficient of determination
nRMSE	Normalized Root Mean Square Error
SMOGN	Synthetic Minority Over-Sampling Technique for Regression with Gaussian Noise
DA	Data Augmentation
GA	Genetic Algorithm
SVM	Support Vector Machine
RFC	Random Forest Classification
MLP	Multi-Layer Perceptron
